# The Utility of Whole Body Computed Tomography in Trauma Activations and the Impact of Incidental Findings on Patient Management: A Review

**DOI:** 10.7759/cureus.72798

**Published:** 2024-10-31

**Authors:** Steven P Green, Salsabeal Al-Saedy, Evan C Thomas, Jacob Glaser

**Affiliations:** 1 Medicine, Elson S. Floyd College of Medicine, Washington State University, Spokane, USA; 2 Surgery, Elson S. Floyd College of Medicine, Spokane, USA; 3 Trauma Surgery, Providence Regional Medical Center, Everett, USA; 4 Trauma Surgery, Madigan Army Medical Center, Joint Base Lewis-McChord, USA

**Keywords:** acute care surgery and trauma, acute trauma care, follow up in critical care, full-body computed tomography, general trauma surgery, incidental finding, incidentaloma, pan scan, post-surgical follow-up, whole-body ct scan

## Abstract

Traumatic injury remains a leading cause of death globally, necessitating rapid and accurate diagnostic tools in emergency settings. Whole-body computed tomography (WBCT) has emerged as an increasingly utilized component in trauma care due to its speed and diagnostic precision. This review summarizes current data on the impact of WBCT on trauma mortality and examines the frequency and clinical implications of incidental findings (IFs). PubMed/Medline was searched for randomized controlled trials (RCT) and meta-analyses in the context of WBCT's effect on trauma mortality as well as retrospective studies investigating the clinical impact of IF. Studies were excluded if they did not measure endpoints of mortality in relation to WBCT or the incidence and impact of IF in WBCT patients. Emergency Department (ED) length of stay (LOS) was significantly reduced by a mean of nine to 34 minutes in WBCT cohorts, with no significant change in hospital or intensive care unit LOS. Contrary to some previous analyses, survival benefit was not reproduced in an RCT in this context. IF were identified in 40-54.8% of WBCT trauma patients, with 29-69.2% of all findings detected on abdominal/pelvic scans. 5.8-31.3% of IF required urgent management. Follow-up on IF was often inadequate and approximately half of cases were omitted from discharge reports. WBCT potentially enhances trauma care through rapid diagnosis and reduced ED LOS. The lack of corroborating RCT evidence highlights the need for additional trials. Effective management of IF remains a critical area for improvement that may optimize patient outcomes. Institutions may benefit from developing guidelines to address the reporting and follow-up of IF in trauma patients.

## Introduction and background

**Figure 1:**
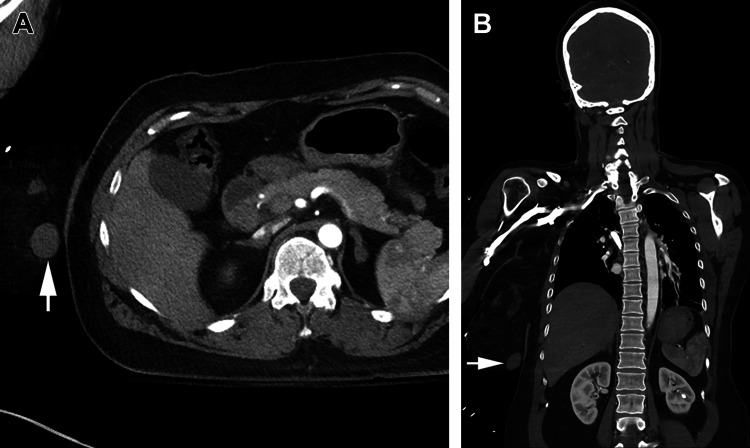
Incidental breast mass Axial (A) and coronal (B) CT images showing an incidental right breast mass (arrows) identified on WBCT. Original image available from analysis by Gunn et. al published in Radiologic Clinics of North America [7]. CT, computed tomography; WBCT, whole-body computed tomography

Traumatic injury remains a leading cause of death worldwide [1]. Computed tomography (CT) has become an indispensable tool for a wide variety of clinicians due to the speed, precision, and diagnostic accuracy provided [2]. A dedicated machine has become an increasingly common sight in the high-volume Emergency Department (ED), and trauma teams have long been utilizing selective CT imaging based on physical findings and mechanism of injury to guide rapid intervention [3]. Delays in timely diagnosis of traumatic injuries measured in minutes can have potentially life-altering implications in the acute setting of trauma care [4]. Whole-body computed tomography (WBCT) trauma protocols seek to quickly identify all life-threatening injuries without reliance on exam-directed imaging to decrease the time to intervention [5]. The clinical benefit of WBCT in trauma patients is still currently debated [6]. Incidental findings (IF) are commonly identified on WBCT, as seen in the example shown in Figure 1. The clinical impact of these IFs has not been well characterized [7]. This review seeks to summarize current data on the effect of WBCT on trauma mortality and consolidate available information surrounding IF identified in these cases.

## Review

Methods

**Figure 2:**
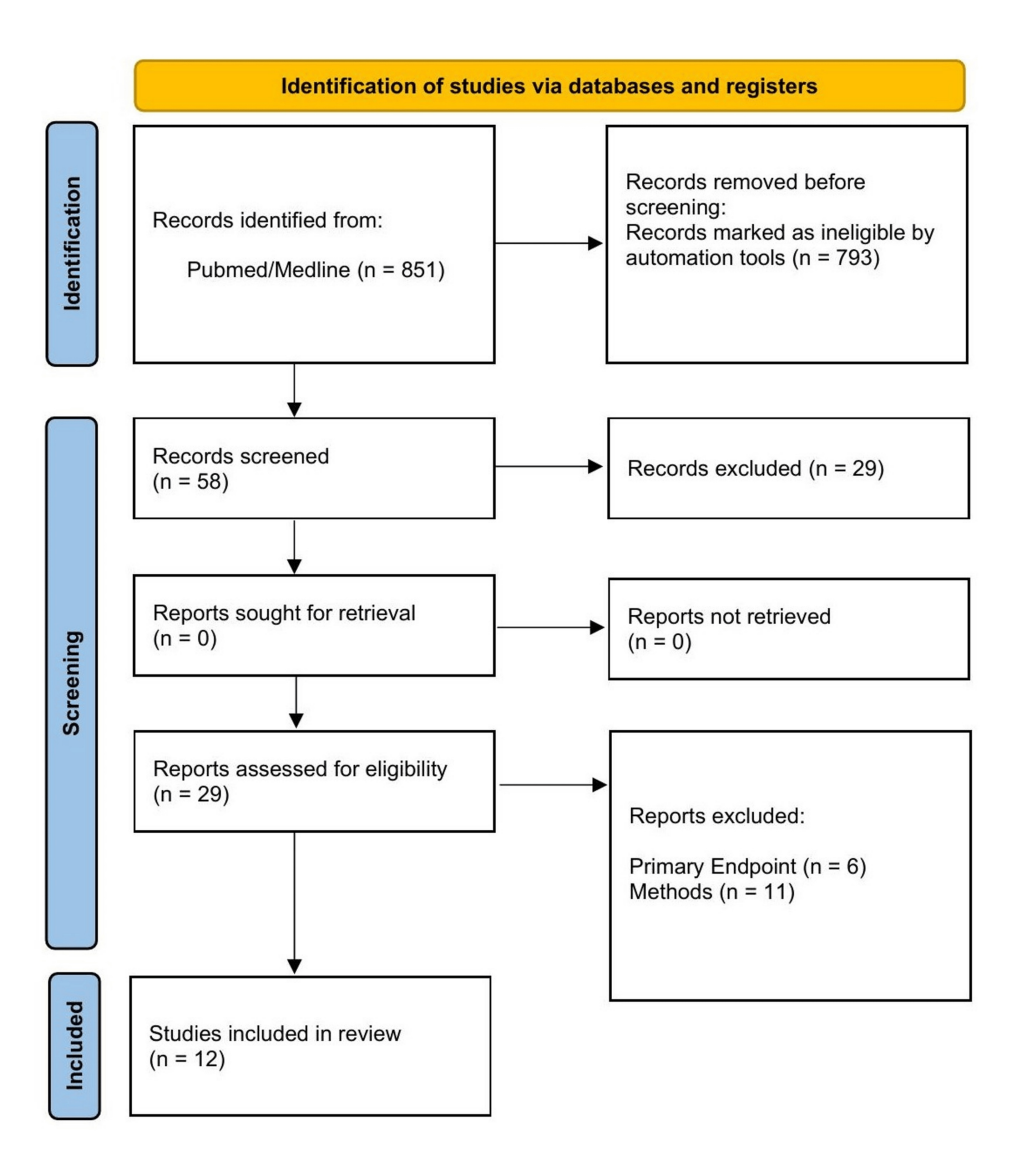
Study selection

Pubmed/Medline was queried using the search terms, “whole body CT trauma incidental,” “full body CT trauma incidental,” and “whole body CT trauma.” Studies published in the last 10 years were considered, detailed in Table 1. Randomized controlled trials (RCT) and meta-analyses evaluating the effect of WBCT on trauma mortality were included. Due to a lack of available meta-analysis or systematic review on the subject, salient retrospective analyses were included presenting data on WBCT IF. Studies were excluded if they did not directly evaluate mortality using WBCT or the incidence and impact of IFs in trauma CT. The study selection process is seen in Figure 2. WBCT is defined as a CT head, neck, chest, and abdomen/pelvis. IFs are defined as any previously unknown non-traumatic finding documented on radiology reports that may or may not impact clinical care. Selective imaging is any physical exam- or history-directed CT in a standard-of-care trauma work-up. Mean statistics are reported as the range of averages collated from included analyses.

**Table 1:** Study characteristics RCT, randomized controlled trial; WMD, weighted mean difference; ICU, intensive care unit; ED, emergency department; LOS, length of stay; WBCT, whole-body computed tomography; CT, computed tomography

Author	Title	Population	Publication	Key Findings
Andrawes et al. [1]	CT scan incidental findings in trauma patients: does it impact hospital length of stay?	N=957. Retrospective analysis	BMJ Trauma Surgery and Acute Care, September 2017	40% of trauma patients had IF on CT. 63% non-urgent f/u. Patients age with IF was much higher vs without, 62.0±22.3 years vs. 49.2±29.5 years (p=0.0001), LOS: 8.7±12.8 days in the IF group, and 6.4±7.7 days by the non-IF group (p=0.0012).
Sierink et al. [2]	REACT-2 study group. A multicenter, randomized controlled trial of immediate total-body CT scanning in trauma patients	N=5,475. RCT	Lancet, August 2016	In-hospital mortality did not differ WBCT (86 (16%) of 541) vs. standard work-up (85 (16%) of 542; p=0.92). In-hospital mortality did not differ between polytrauma WBCT (81 (22%) of 362) vs. standard work-up (82 (25%) of 331; p=0.46).
Chidambaram et al. [3]	A meta-analysis of the efficacy of whole-body computed tomography imaging in the management of trauma and injury	11 studies N=32,207. Meta-analysis	Injury, August 2017	WBCT cohort had lower overall (OR=0.79; 95% CI 0.74,0.83, p<0.05) and 24h mortality rates (OR=0.72, 95% CI 0.66,0.79, p<0.05). WBCT arm had a shorter ED stay (MD=-14.81; 95% CI -17.02, -12.60, p<0.00001).
Jiang et al. [4]	Comparison of whole-body computed tomography vs selective radiological imaging on outcomes in major trauma patients: a meta-analysis	11 trials, N=26,371. Meta-analysis	Trauma, Resuscitation and Emergency Medicine September 2014	WBCT was associated with a lower mortality rate (pooled OR: 0.66, 95% CI: 0.52 to 0.85). The WBCT group had a shorter stay in the ED WMD, −27.58 min; 95% CI, −43.04 to −12.12).
Fathi et al. [5]	Diagnostic utility of whole-body computed tomography/pan-scan in trauma: a systematic review and meta-analysis study	27 studies, N=68,838. Meta-analysis	Journal of Emergency Radiology, April 2024	WBCT protocol is associated with 2% reduction in mortality compared with selective imaging (odds ratio: 0.94). Mean age: 45.0±24.7.
Arruza et al. [6]	Systematic review and meta-analysis of whole-body computed tomography compared to conventional radiological procedures of trauma patients	14 studies, N=63,529. Meta-analysis	European Journal of Radiology, August 2020	WBCT was found to reduce ED time (SMD=-0.709, CI: -1.198 to -0.220, p=0.004). Patients experienced a similar 24 h mortality rate (p=0.450), and hospital (p=0.541) and ICU LOS (p=0.457).
Gunn et al. [7]	Improving Outcomes in the Patient with Polytrauma: A Review of the Role of Whole-Body Computed Tomography	6 studies, N=20,582. Meta-analysis	Radiologic Clinics of North America, July 2017	WBCT is associated with improved or no change in patient survival and reduces ED LOS. IF abdominopelvic prevalence from 12% to 45%.
Caputo et al. [8]	Whole-body computed tomographic scanning leads to better survival as opposed to selective scanning in trauma patients: a systematic review and meta-analysis	7 Studies, N=25,782. Meta-analysis	Journal of Trauma and Acute Care Surgery, October 2014	Mortality rate was significantly lower for WBCT vs. selective scanning (16.9; 95% CI, 16.3-17.6 vs. 20.3; 95% CI, 19.6-21.1, p<0.0002, respectively). The pooled odds ratio for mortality rate was 0.75 (95% CI, 0.7-0.79), favoring WBCT.
Fakler et al. [9]	Retrospective analysis of incidental non-trauma associated findings in severely injured patients identified by whole-body spiral CT scans	N=704. Retrospective analysis	Patient Safety in Surgery, August 2014	43% rate of IF. 6.7% had high clinical relevance altering management. IFs were located in the head in 11.2% of patients, 6.1% in the neck, 20.7% in the thorax, 49.4% in the abdomen, and 12.3% in the pelvis.
Kumada et al. [10]	Incidental findings on whole-body computed tomography in trauma patients: the current state of incidental findings and the effect of implementation of a feedback system	N=199. Retrospective analysis	Acute Medicine and Surgery, July 2019	WBCT revealed IF in 40.1% of patients. IF recognized at a rate of 23.3% by radiology. Mean age with IF was 62.8±19.5 years, IF in liver/gallbladder was 27.8%, kidney: 21.5%, lung: 17.7%, intracranial area: 16.5%.
Liu et al. [11]	Incidental Findings on Whole-body Computed Tomography in Major Trauma Patients: Who and What?	N=217. Observational	The American Surgeon, February 2021	Abdominal IF: 69.2%, head/neck: 17.3%, and chest: 13.5%. 31.3% of IF required further treatment. Mean age of IF altering treatment was 57.3 vs. 38.9 (P<0.001).
Seah et al. [12]	Incidental findings on whole-body trauma computed tomography: Experience at a major trauma centre	N=104. Observational	Injury, March 2016	54.8% had IF. 5.8% of IF altered care due to severity. 41.3% of IF were not clinically relevant. The incidence of IF increases with age.

Findings

The REACT-2 trial is the only RCT evaluating mortality in WBCT trauma patients to the best of this author's knowledge. At completion, Sierink et al. found no clinically significant difference in in-hospital mortality between the WBCT group and selective imaging [2]. Meta-analysis performed by Arruzza et al. and Gunn et al. supports this conclusion [6,7]. Conflicting the RCT findings, multiple meta-analyses have also been published showing significantly lower mortality (reported OR=0.67-0.79) for WBCT cohorts [3-5,8]. Mortality was measured within 24 hours or as a pooled odds ratio [2-6,8]. ED LOS was consistently found to be reduced by a mean of nine to 34 minutes by utilizing a WBCT protocol [2-7]. Hospital LOS was primarily found to have no significant change in duration, despite some authors reporting a significant reduction [2-7]. ICU LOS was found to have no significant change in duration between WBCT and selective imaging cohorts [3,4,6]. Patients in WBCT groups are more critically injured as determined by injury severity score (ISS) than those receiving selective imaging [2-8]. 

**Figure 3:**
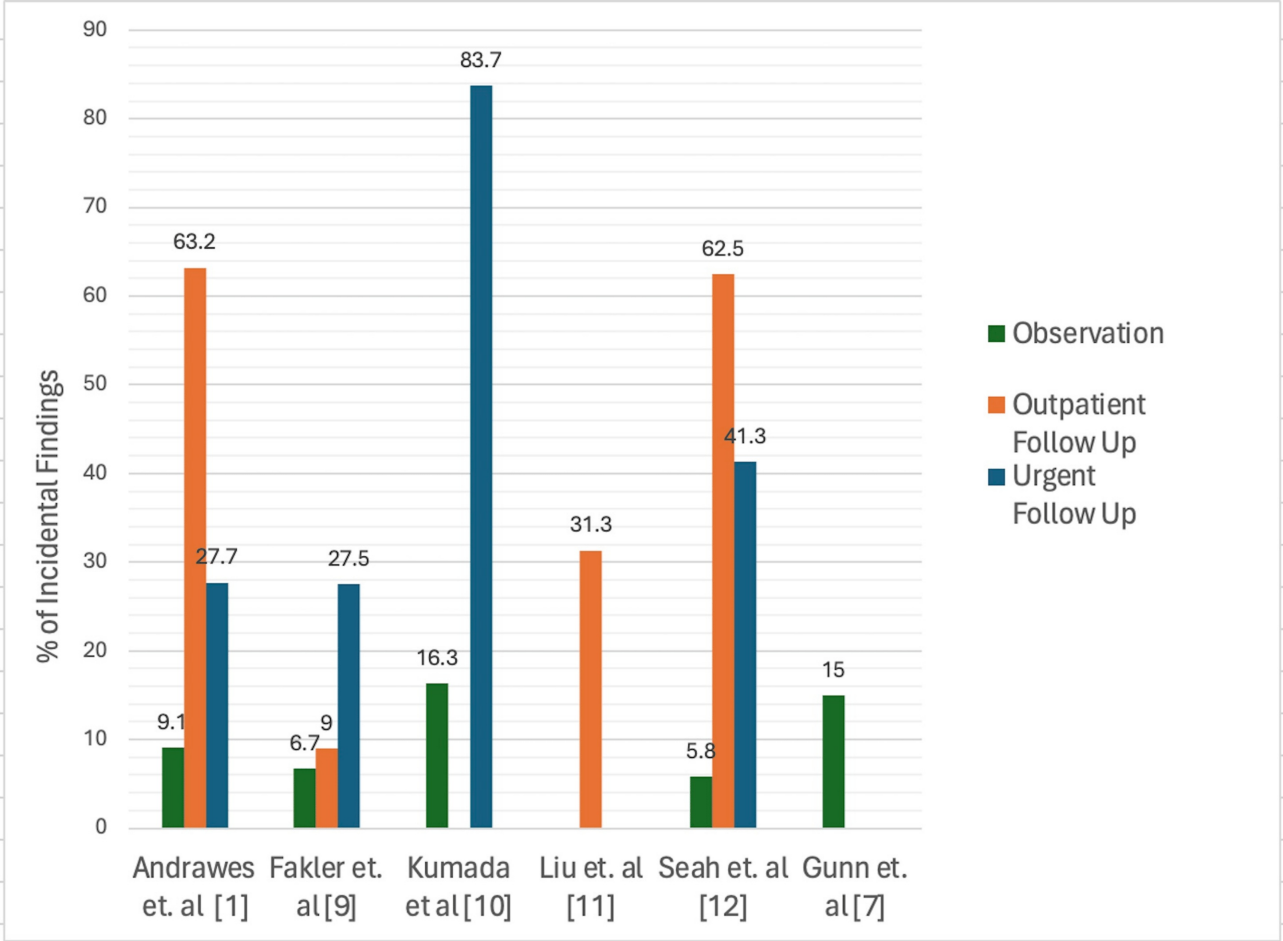
Stratification of IFs by clinical severity IF, incidental finding

**Figure 4:**
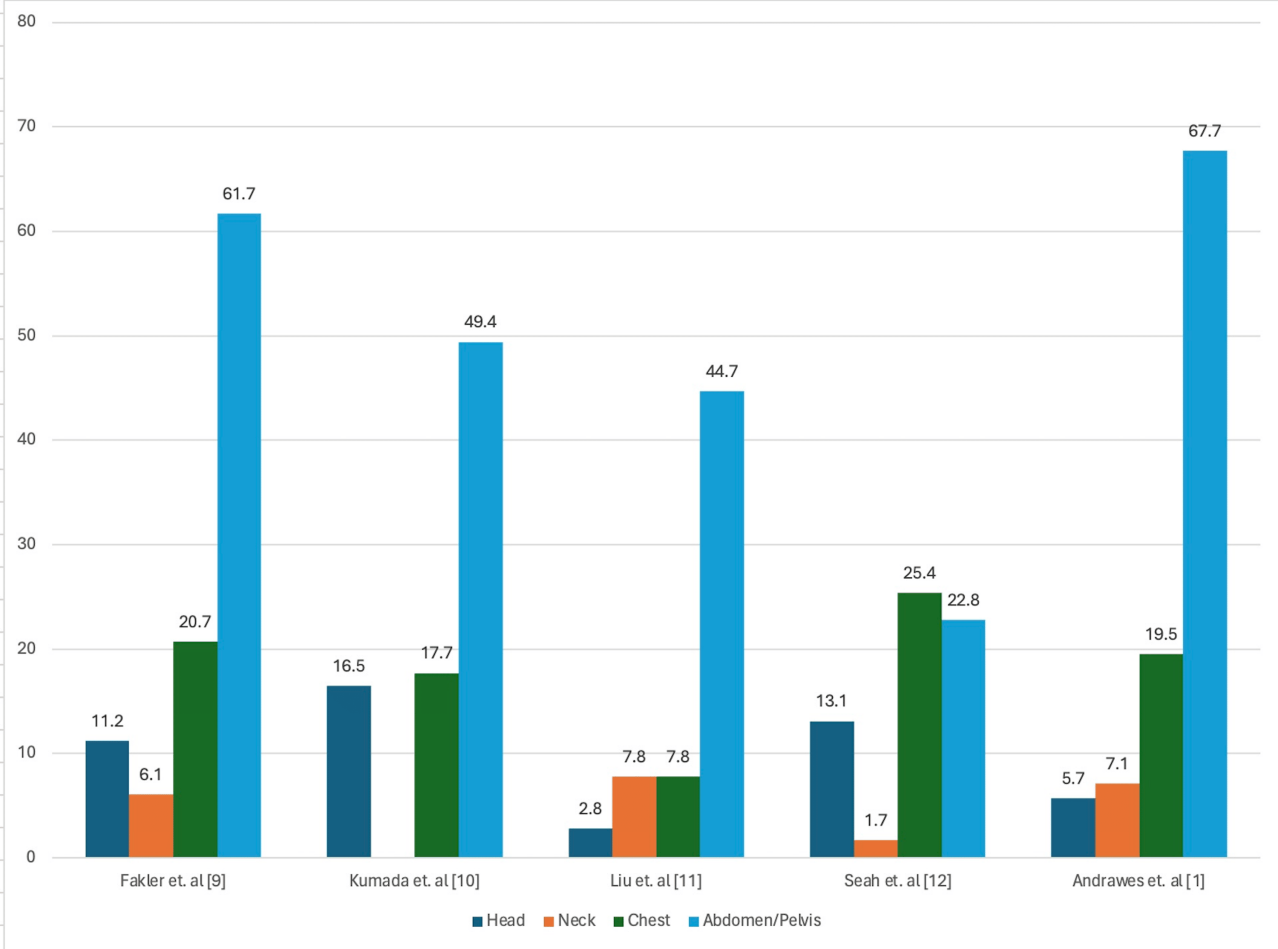
Percentage of IF by anatomical region IF, incidental finding

IFs are poorly characterized in the literature found during this review. A summary of specific findings is displayed in Table 2. Males represented an outsized proportion of trauma patients and were identified more frequently with an IF [1,9-11]. The rate of IF increased with age [1,11]. The mean age of patients with IF was 57.3 to 62.8 years [1,10,11]. Reported occurrence of IF ranges from 40.0% to 54.8% in WBCT trauma populations with the majority of findings requiring follow-up, as illustrated in Figure 3 [1,3,9-12]. The abdominal/pelvic CT was consistently identified as containing the highest amount of IF, ranging from 29% to 69.2% of all findings; proportions of IF by anatomic region are visible in Figure 4 [3,5,9-12]. Three studies evaluated the LOS of patients with IF versus those without, finding the duration of hospital stay to remain similar or increase compared to controls [1,5,10]. The most clinically severe IF were reported at a rate of 5.8-31.3% [1,9-12]. Regardless of severity, IFs are poorly communicated through referral and discharge reports [9-11]. Kumada et al. made the observation that IFs were only reported on initial radiology in approximately 23.3% of cases [10]. Fakler et al. found that of the most clinically important IF, the results were not included or inadequately addressed on 47.1% of discharge reports [9]. Liu et al. and Gunn et al. also describe 20% to 48.4% rates of follow-up on these findings [7,11]. A concerning data point from Liu et al. found that of WBCT trauma patients needing follow-up for IF, 63.2% had no record of follow-up and 10.7% died before findings were addressed [11].

**Table 2:** Summary of specific IFs IF, incidental finding

Head, Neck, and Spine				
IF	Seah et al. [12]	Liu et al. [11]	Andrawes et al. [1]	Total
Arachnoid cyst	3	2	3	8
Hydrocephalus	2	0	0	2
Porencephaly	0	1	0	0
Pneumocephaly	1	0	0	1
Small vessel disease	2	0	0	2
Acute infarct	1	0	0	1
Prior brain infarct	1	0	0	1
Retroclival hematoma	1	0	0	1
White matter changes	1	0	0	1
Brain lesion	0	0	11	11
Brain mass	0	0	1	1
5^th^/6^th^ ventricle	0	2	0	2
Carotid artery calcification	1	0	0	1
Vertebral artery aneurysm	0	0	1	1
Ethmoiditis	1	0	0	1
Mastoiditis	1	0	0	1
Foreign body	1	0	0	1
Sinus polyp	0	1	17	18
Thyroid nodule	1	10	39	50
Spondylosis	1	14	0	15
Disc prolapse	2	0	0	2
Degenerative spinal changes	0	10	7	17
Compression fracture	3	0	0	3
Pars defect	1	0	0	1
Diffuse hyperostosis	1	0	0	1
Congenital fusion	1	0	0	1
Hypoplastic 1^st^ rib	1	0	0	1
Ligament ossification	1	0	0	1
Vertebral deformity (0)(2)	0	2	0	2
Lumbar lipoma (0)(1)	0	1	0	0
Thoracic				
Aberrant subclavian artery	0	1	0	1
Subclavian artery aneurysm	0	0	1	1
Kommerell diverticulum	0	2	0	2
Bovine aortic arch	0	0	3	3
Left-sided SVC	0	0	2	2
Aortic dissection	0	1	0	1
Atherosclerotic disease	5	0	0	5
Large vessel thrombus	3	0	0	3
Coronary artery calcification	2	0	0	2
Mediastinal lymphadenopathy	1	0	14	15
Mediastinal mass	0	0	2	2
Lung mass	0	0	6	6
Emphysema	8	0	0	8
Pulmonary nodules	4	6	70	80
Pleural nodule	1	0	2	1
Pleural scarring	1	0	0	1
Pulmonary emboli	1	0	0	1
Pulmonary fibrosis	1	0	0	1
Pulmonary bullae	0	2	0	2
Ventricular hypertrophy	1	0	0	1
Cardiomegaly	1	0	0	1
Esophageal diverticulum	0	2	0	2
Diaphragmatic hernia	0	0	2	2
Hiatal Hernia	0	1	10	11
Rib pseudoarthrosis	0	1	0	0
Bone cyst	0	2	0	2
Vertebral hemangioma	0	1	0	1
Abdomen and pelvis				
Abdominal aortic aneurysm	0	0	11	11
Iliac artery aneurysm	0	0	3	3
Liver nodule	0	5	0	5
Hypodense liver lesion	0	0	30	30
Dilated intrahepatic ducts	0	0	5	5
Hepatic Hemangioma	0	2	4	6
Hepatic cyst	1	17	19	37
Pancreatic tumor	2	0	0	2
Hypodense pancreatic lesion	0	0	15	15
Pancreatic cyst	0	0	5	5
Atrophic pancreas	1	0	0	1
Splenic hemangioma	0	0	13	13
Splenic cyst	5	0	1	6
Accessory spleen	0	0	3	3
Splenomegaly	0	0	1	1
Retroaortic left renal vein	1	0	0	1
Renal cyst	5	20	91	116
Renal calculi	0	7	13	20
Polycystic kidney	0	4	0	4
Hydronephrosis	0	1	0	1
Duplicate renal system	0	0	4	4
Horseshoe kidney	0	0	3	3
Hypodense renal lesion	0	0	36	36
Renal artery aneurysm	0	0	1	1
Adrenal myolipoma	0	0	2	2
Adrenal tumor	0	3	0	3
Adrenal cyst	0	1	0	1
Adrenal nodule	0	0	36	36
Bile duct tumor	0	1	0	1
Gallstones	0	13	4	17
Small bowel dilation	1	0	0	1
Gastric mass	0	0	1	1
Mesenteric mass	0	0	1	1
Colon lesion	0	0	2	2
Diverticular disease	4	4	5	0
Mesenteric lymphadenopathy	2	0	0	2
Bladder mass	0	0	1	1
Bladder diverticulum	0	0	7	7
Hernia	2	3	8	13
Undescended testicle	0	0	1	1
Hydrocele	0	0	3	3
Prostate enlargement	0	0	4	4
Prostate tumor	0	2	0	2
Pelvic mass	0	0	2	2
Uterine fibroid	0	0	5	5
Endometrial thickening	0	0	1	1
Endometrial mass	0	0	3	3
Adnexal teratoma	0	1	0	1
Ovarian cyst	2	1	20	23

Discussion

There is currently a significant body of evidence that shows WBCT provides a reduction in mortality despite a higher baseline ISS than patients with selective imaging [3-5,8]. However, the absence of key confirmatory findings provokes some consideration of previous results [2,6,7]. Generalization of these findings must be considered carefully with respect to the method of data collection. The nature of traumatic injury is such that prospectively selecting patients presents considerable challenges [8]. As a result, the majority of data collected to date for mortality analysis is retrospective or observational in nature. Additionally, the heterogeneity of data may potentially obfuscate reporting results with the greatest accuracy. As noted by Sierink et al., level 1 evidence is lacking despite the suggested clinical benefit of WBCT found in other studies [2-5,8]. 

50-60% of trauma deaths are thought to take place in the “golden hour” after injury, and the rapid initiation of treatment during this time has long been a pillar of trauma care [6]. Despite WBCT providing rapid and comprehensive injury identification, conflicting mortality data suggests it is still unclear as to whether life-threatening injuries are more accurately identified versus selective imaging. A significant reduction in ED LOS for WBCT patients was consistently found during this review. It seems likely that rapid whole-body imaging facilitates faster diagnosis and reduces the time from injury to treatment. That this did not manifest as a survival benefit in the REACT-2 trial suggests there may be variables that are not accounted for [2]. Selection bias for patients stable enough to undergo WBCT may be one confounding element. The most critically ill patients are more likely to be taken straight to the operating room or expire before WBCT is performed. Another potential explanation for the discordance is the use of sequential selective scanning that comprises a WBCT equivalent. It is unclear how often congruent selective imaging results in WBCT equivalent imaging; however, Sierink et al. reported that 46% of selective imaging patients received sequential segmental components of WBCT. If equivalent rates are occurring in other centers, then this analogous imaging could conceivably partially account for similar rates of mortality. Trauma mortality remains a crude metric for success. Time to treatment, amount of missed injury, or reduction in sequelae may provide more insight into the most successful treatment strategies.

Further obscuring the accurate reporting of mortality is the use of the ISS to match WBCT cohorts with comparators. ISS is not calculated in real time. A retrospective value of ISS is applied to patients after assessment and imaging have located the extent of the injury. It is not a clinical tool that determines imaging strategy. The comprehensive nature of WBCT provides the capability to rapidly identify all injuries, including those that are not yet clinically apparent as well as those which may not require immediate treatment [2]. Early detection may be reflected in the beneficial survival outcomes [3-5,8]. Alternatively, this may be viewed as a mechanism that inflates the clinical severity and influences mortality statistics. 

Unsurprisingly, WBCT imaging has led to a high level of IFs. In the literature utilized in this review, it was more common to broadly categorize the type, location, or clinical severity of an IF rather than quantifying specifics. Importantly, this method of grouping data facilitates broader generalizations surrounding the need for follow-up, and the proportion of findings reaching a threshold to alter patient management. However, this results in few specific findings to draw from when considering what IF are driving changes in clinical behavior. Specific IF summarized in this review covers an extremely wide breadth from not clinically significant, to life-altering malignancy, to urgent vascular aneurysm. It should be noted that these specific findings were available in only three studies included in this review and represent a small collective sample size of n=1,278 [1,11,12]. A greater degree of information was available concerning the implications of IF. 

Follow-up on these findings was deemed necessary in nearly half of the studied population. This review has demonstrated that a proportion of IF does warrant rapid intervention [7-11], even among subgroups that were found to sustain much less critical traumatic injury [1,7-11]. Communication and follow-up of these findings were poor when measured [7,9-11]. It is unclear if these IFs were determined not to be significant by the treating physician, continuity of care was disrupted when transferring care, or another unspecified reason. Presently, it seems likely that there is an opportunity to improve communication and follow-up on IF in this population. A protocol using a natural language processing-based algorithm can trigger follow-up comments in CT reads without adding burden to time-sensitive imaging reads. Additionally, the protocol to include follow-up information in tertiary survey notes represents another area where this communication may be integrated.

The risks of radiation, financial cost, and resource utilization are all valid concerns for the evaluation of a clinically silent imaging finding. The desire for comprehensive care, due diligence, or unease with medico-legal consequences that may result from not following up prompts many clinicians to further investigate an IF. Currently, the scant data available and the absence of guidelines surrounding IF are likely allowing some patients to slip through the cracks and may prompt unnecessary investigation for others. Further discussion in the community will require additional research to achieve consensus. In the interim, there may be a benefit to some form of institutional guidance on the reporting and evaluation of IF.

Limitations

This review has several limitations. Data used in the included studies is largely retrospective or observational with a poorly defined degree of heterogeneity. Gender was not distributed equally among studies examining IF, heavily skewing toward the male population with one study failing to report demographic data. The categorization of IF is not uniformly defined and individual authors subjectively determine the severity and clinical significance, allowing the risk of bias. Study selection for this review was performed using limited search criteria for the most salient information. Although efforts were made to reduce bias, the possibility of selection bias cannot be excluded.

## Conclusions

ED LOS was significantly reduced in WBCT cohorts. Retrospective and observational data suggest a reduction in trauma mortality associated with WBCT use. However, the REACT-2 trial, the only RCT to date, did not find a clinically significant difference in mortality between WBCT and selective imaging cohorts. It is unclear how ISS-dependent evaluation and contiguous selective scanning may be influencing these outcomes. This discrepancy highlights the need for further research, particularly RCTs, to further define the clinical utility of WBCT. Future research may benefit from evaluating more nuanced metrics of clinical outcomes after trauma in addition to mortality when investigating this topic. IFs are common in trauma patients undergoing WBCT, necessitating follow-up in nearly half of cases. Abdominal scans are the most frequent source of these findings. Despite their prevalence, communication and follow-up on IF are frequently inadequate. Institutions may benefit from implementing internal protocols to ensure important findings are communicated promptly and addressed appropriately. WBCT allows for rapid detection of traumatic injuries, yet a definitive answer as to how this affects patient outcomes remains elusive. The integration of WBCT into trauma care protocols may benefit from a comprehensive approach that addresses both immediate injuries and, although not the intended goal of the trauma assessment, undiagnosed health conditions. Future studies should aim to provide more robust evidence to guide clinical practice surrounding the management of IF and optimize the use of WBCT in trauma care.
